# Risk Factors and Immunoinflammatory Mechanisms Leading to Atherosclerosis: Focus on the Role of Oral Microbiota Dysbiosis

**DOI:** 10.3390/microorganisms11061479

**Published:** 2023-06-01

**Authors:** Riccardo Mattia Ricciardi, Alessia Cipollone, Damiano D’Ardes, Davide Di Giacomo, Pamela Pignatelli, Francesco Cipollone, Maria Cristina Curia, Paolo Magni, Marco Bucci

**Affiliations:** 1 Department of Medicine and Aging Sciences, Università degli Studi “Gabriele d’Annunzio” di Chieti-Pescara, 66100 Chieti, Italy; riccardomattiaricciardi@gmail.com (R.M.R.); acipollone@live.it (A.C.); davide.digiacomo95@gmail.com (D.D.G.); francesco.cipollone@unich.it (F.C.); mbucci@unich.it (M.B.); 2 Regional Center for the Study of Atherosclerosis, Hypertension and Dyslipidemia, “SS Annunziata” Hospital—ASL, 66100 Chieti, Italy; 3 COMDINAV DUE, Nave Cavour, Italian Navy, Stazione Navale Mar Grande-Viale Jonio, 74122 Taranto, Italy; pamelapignatelli89p@gmail.com; 4 Department of Medical, Oral and Biotechnological Sciences, Università degli Studi “Gabriele d’Annunzio” di Chieti-Pescara, 66100 Chieti, Italy; mariacristina.curia@unich.it; 5 Epidemiology and Preventive Pharmacology Service (SEFAP), Department of Pharmacological and Biomolecular Sciences, Università degli Studi di Milano, 20133 Milan, Italy; paolo.magni@unimi.it; 6 Department of Pharmacological and Biomolecular Sciences “Rodolfo Paoletti”, Università degli Studi di Milano, 20133 Milan, Italy; 7 IRCCS MultiMedica, Sesto S. Giovanni, 20099 Milan, Italy

**Keywords:** atherosclerosis, *Porphyromonas gingivalis*, *Fusobacterium nucleatum*, cardiovascular disease, heterozygous familial hypercholesterolemia, secondary cardiovascular prevention

## Abstract

Cardiovascular diseases (CVD), including myocardial infarction and stroke, are currently the leading cause of morbidity, disability and mortality worldwide. Recently, researchers have focused their attention on the alterations of the gut and oral microbiota, investigating the possible role of their dysbiosis in the pathogenesis and/or progression of CVD. In this regard, it has been shown that endothelial dysfunction, a major feature of CVD, can also be induced by chronic periodontal infection, due to a systemic pro-inflammatory condition, as suggested by increased plasma levels of acute phase proteins, IL-6 and fibrinogen. Moreover, proatherogenic dysfunctions can also be promoted by direct bacterial invasion of the endothelium. This review reports the current evidence about the possible role of oral microbiota dysbiosis and the related immunoinflammatory components in the pathophysiology of atherosclerosis and associated CVD. It is concluded that integration of oral microbiota sampling into clinical practice may result in a more accurate assessment of CV risk in patients and even modify their prognosis.

## 1. An Introduction to Atherosclerotic Cardiovascular Diseases: Epidemiology and Role of Associated Risk Factors

**Figure 1:**
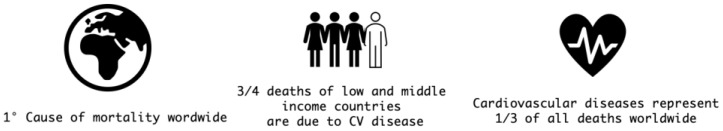
Cardiovascular diseases as leading cause of mortality worldwide.

Cardiovascular diseases (CVD) are currently the leading cause of mortality worldwide and a major cause of morbidity and disability, and are mainly manifested by myocardial infarction and stroke, according to WHO data [1], as represented in Figure 1. These diseases recognize atherosclerosis and thrombosis as their main pathogenetic events. Atherosclerosis-related CVD (ASCVD) results from well-established risk factors such as arterial hypertension, diabetes mellitus (DM), visceral obesity, dyslipidemia and age, as well as unhealthy lifestyle habits, such as cigarette smoking, inappropriate nutrition and sedentarity [2]. Overall, cardiovascular risk (CVR) is defined as the set of factors showing high correlations between expected and observed mortality [3] in each group of individuals presenting these factors. According to WHO indications, cardiovascular diseases represent the most important cause of mortality, especially in low- and middle-income countries (see Figure 1). CVR factors can be divided into modifiable (arterial hypertension, DM, dyslipidemia, obesity, diet, smoking, alcohol abuse and psychosocial factors) and non-modifiable (age, gender, ethnicity history and genetics) factors, as listed in Table 1 [4].

Age plays a fundamental role in the genesis of ASCVD [5], due to the burden of comorbidities that can be found as life progresses. Some of these comorbidities represent additional risk factors for CVD itself [5], which could also be present independent of aging. In the premenopausal era women are protected from CVD [6] due to the anti-atherogenic action of estrogen hormones [7].

Cigarette smoking is responsible for oxidative stress, inflammation and vascular dysfunction [8], as well as diseases inherent to other systems, primarily the respiratory system. It is responsible for various ASCVDs, such as stable angina, myocardial infarction, sudden cardiac death and aortic or peripheral artery disease [9]. From a pathophysiological point of view, smoking-induced vascular dysfunction is derived from a decrease in the availability of nitric oxide [10], increased ability of the endothelium to recruit immune cells [11] and an increase in the oxidation of circulating LDL cholesterol [12].

CVR may therefore be assessed starting from a careful anamnestic interview and from clinical and instrumental evaluations, in order to obtain an accurate prognostic and therapeutic patient evaluation. The ESC guidelines have given a definition of ASCVD (see Table 2) [13]. Moreover, ESC considers patients at high/very high cardiovascular risk if they have at least one of the following features:

1. History of previous CVD or ASCVD;
2. Type 2 or type 1 DM;
3. Chronic kidney disease (CKD) or chronic renal failure:
4. Very high burden of individual CVR factors.

These patients will, therefore, be taken care of without further need for classification. All patients at lower risk are stratified according to risk charts in the “SCORE” system [14], which estimates the 10-year risk of fatal cardiovascular events in Europeans aged between 40 and 70, based on sex, smoking habits, age, systolic blood pressure, and total cholesterol.

Arterial hypertension, a major cause of CVD, doubles the risk of coronary heart disease, congestive heart failure, ischemic or hemorrhagic stroke, renal failure, and peripheral arterial disease [15]. Hypertension-induced wall stress on the vascular wall predisposes the vessel to atheromatous lesions and accelerates a pre-existing atherosclerotic process, especially at the coronary and cerebral levels [16].

As far as glucose metabolism dysfunctions are concerned, it can be noted that DM, due to excess blood glucose and impaired insulin action, promotes an increase in vascular oxidative stress. When insulin resistance is also present, such as in type 2 DM (T2DM), there is an alteration of the phosphatidyl-inositol 3-kinase (PI3K) pathway, which causes a reduction in a fundamental hemorheologic agent, such as nitric oxide, in favor of endothelin [17]. Insulin resistance in turn correlates with body weight and in particular with the amount of visceral fat mass [18].

Obesity, especially in the visceral compartment, is a risk factor as it determines the onset of a low-grade chronic inflammation conditions [19] which in turn acts as a pathological substrate for arterial hypertension, insulin resistance and lipid imbalance [3]. On the other hand, physical inactivity causes a reduction in lipoprotein lipase activity, in HDL cholesterol and in triglyceride uptake in skeletal muscle, as observed in mouse models that mimic the effects of a sedentary lifestyle [20]. Moreover, it has also been demonstrated that a sedentary condition results in the modulation of gene transcription to promote a dysmetabolic condition in rat skeletal muscle [21].

Dietary patterns associated with current economic developments have led, in countries with higher living standards, to an increased consumption of saturated animal fats and hydrogenated vegetable fats rich in pro-atherogenic trans fatty acids and to an increase in simple carbohydrates [3]. On the contrary, the implementation of the Mediterranean diet with fish, monounsaturated fats from olive oil, fruit, vegetables, legumes and whole grains [22], has been shown to lead to an improvement in blood levels of cholesterol, blood sugar and systolic blood pressure [23]. Lastly, dyslipidemia is a well-defined CVR factor [3]. In particular, the direct correlation of circulating LDL-C levels with ASCVD has been largely demonstrated [24]. Circulating levels of HDL-C, which are inversely correlated with ASCVD [25], are also important in this context.

Atherosclerosis is a chronic disease affecting medium- and large-caliber arteries and shows an immunoinflammatory pathogenesis [26], capable of causing a discrepancy between oxygen demand and supply through a hemodynamic/stenotic, thrombotic or thromboembolic mechanism [27]. From a pathophysiological point of view, the atherosclerotic plaque and the associated thrombosis are the elements responsible for determining the onset of acute cardiovascular or cerebrovascular events, i.e., what is clinically defined as ASCVD [13].

As mentioned above, numerous factors contribute to the pathogenesis of an atherosclerotic lesion and the impact of circulating cholesterol levels, in particular LDL-C [24], in determining low-grade chronic inflammation of the vascular wall [28].

In recent decades, attempts have been made to investigate new factors to be correlated with endothelial inflammation and which may be partially responsible for the so-called “residual cardiovascular risk”. In particular, attention has grown towards the human microbiota, since thrombosed atheromatous plaques of patients undergoing thromboendarterectomy have tested positive for selected microbial agents of the oral or intestinal bacterial populations [29].

Among the risk factors, hypercholesterolemia, and in particular the increase in low-density lipoproteins (LDL-C), is an established causal factor for ASCVD [24]. Atherosclerosis was once considered exclusively a lipid-storage disease, but it is now recognized as a subacute inflammatory condition of the vessel wall characterized by infiltration of macrophages and T cells that interact with endotheliocytes [19].

Morphological and functional studies of the atherogenesis process indicate that the initial lesion is due to the subendothelial accumulation of lipoproteins rich in apolipoprotein B, such as LDL, very low density lipoproteins (VLDL) and Lipoprotein (Lp)(a). This event occurs mainly at the level of arterial branches and bifurcations, which are recognized as the sites with the most turbulent blood flow [30].

Lipoproteins are synthesized in the liver from endogenous or exogenous lipids with the function of transporting fatty acids, triglycerides and cholesterol in the blood and distributing them to peripheral tissues [31]. VLDLs are produced by the liver, which, by progressively releasing triglycerides to the tissues, are first converted into intermediate-density lipoproteins (IDL) and then into LDLs, that contain cholesterol esters [32]. Moreover, lipids introduced into the diet are released into the bloodstream by the enterocytes as chylomicrons, from which, with the progressive release of triglycerides, the so-called remnants are generated. These are triglyceride- and cholesterol-rich lipoproteins with highly atherogenic properties [32]. Lp(a) is chemically similar to LDL and, similarly to this, appears to have atherogenic potency by itself [33].

Finally, there are several high-density lipoproteins (HDL) which are rich in cholesterol, important for the reverse transport of cholesterol and responsible for clearing the tissues from excess cholesterol [34].

We can therefore distinguish lipoproteins on the basis of their content and of the surface proteins (apolipoproteins) that characterize them.

CVD susceptibility reaches a high level of severity in patients with specific genotypes, such as in familial hypercholesterolaemia (FH), resulting in lifelong elevations of LDL-C levels. FH is an autosomal dominant disorder caused by mutations in the gene encoding low-density lipoprotein receptor (LDLR), apolipoprotein B (ApoB) or proprotein convert subtilisin/kexin type 9 (PCSK9). Carriers of these mutations, even if heterozygous (HeFH), have a much higher ASCVD risk due to the long-lasting LDL-C burden [35,36]. Another high-risk cohort includes individuals with polygenic hypercholesterolemia, consisting of a series of mutations (single nucleotide polymorphisms, SNPs) that individually have a minor impact, but which, when added together, can increase circulating levels of LDL-Cs. The cumulative impact of these SNPs can be quantified by calculating an LDL-C SNP score [37]. Interestingly, susceptibility to ASCVD differs among these patients, even when LDL-C levels are similar [38], suggesting that a pathogenetic role for ASCVD may be played by other factors. On the other hand, most individuals with proven atherosclerosis in the USA have normal blood lipids, although these “reference values” in industrialized countries may actually exceed the true human baseline (as data from agricultural societies suggest) [39].

## 2. Low-Grade Chronic Inflammation and Atherothrombosis: Clinical Evidence and Molecular Pathophysiology

In a very important study conducted by Ross and colleagues [40], atherosclerosis was described for the first time as an inflammatory disease, not just the result of the accumulation of lipids. The cellular and molecular mechanisms involved in atherogenesis do not differ from those of chronic inflammatory or fibroproliferative diseases such as rheumatoid arthritis or glomerulosclerosis [40].

Various studies have investigated whether plasma markers of inflammation can have prognostic, if not therapeutic, implications for the atherosclerotic process, as clearly shown by the current medical intervention plans for the progression of atherosclerosis aimed at reducing plasma cholesterol levels [41].

C reactive protein (CRP) has been identified as the best marker of inflammation, in the face of poor specificity [42]. A high-sensitivity diagnostic test for CRP (hsCRP—High Sensitivity C Reactive Protein) has been developed that is able to measure levels of the protein slightly above the reference values in a low-grade chronic inflammatory context [43]. Several studies have shown that an increase in hsCRP is associated with an increased risk of cardiovascular events, which could suggest its use as a predictor of clinical events independently of cholesterol levels [44]. However, other studies have failed to confirm any relationship between low-grade chronic inflammation detected by hsCRP and increased CVR [45,46]. Nonetheless, in some guidelines, hsCRP is included among the prognostic factors, especially in intermediate-risk patients [47].

The JUPITER trial in 2008 evaluated the effect of rosuvastatin on CVR in non-dyslipidemic primary-CV-prevention patients with high hsCRP [48], since there is strong experimental evidence in favor of the anti-inflammatory power of statins [49]. This study enrolled healthy men and women with maximal LDL values of 130 mg/dL and hsCRP greater than 2 mg/L. They were given 20 mg/day rosuvastatin or a placebo as a control. The primary endpoints were myocardial infarction, stroke, arterial revascularization, hospitalization for unstable angina, or death from cardiovascular causes. After an average follow-up of 23 months, the study was stopped: Compared to placebo, LDL levels decreased by 50%, hsCRP levels decreased by 37% compared to the placebo and mortality was significantly improved against primary endpoints, with a cumulative incidence of 0.77 in the statin group and 1.36 in the control group (HR of 0.56 for statin with 95% CI, 0.46 to 0.69, *p*-value < 0.00001) [48].

In the context of the modulation of the inflammatory component of atherosclerosis, in 2017 the CANTOS clinical trial aimed to evaluate the impact of an anti-inflammatory treatment on CVR, rather than just on lipid-lowering. In this study, ten thousand patients with a previous myocardial infarction and elevated hsCRP (>2 mg/dL) were treated with different dosages of the anti-interleukin (IL)-1β monoclonal antibody canakinumab versus placebo. After 48 months, the group that enjoyed the greatest cardiovascular benefits was the group with a daily intake of 150 mg monoclonal antibody [41], which showed a median reduction in hsCRP of 37%, and an incidence of CV events of 3.86/100 person years versus 4.50/100 person years in the placebo (hazard ratio of 0.85 versus the placebo). The lipid profile did not undergo changes due to the tested treatment [41].

Therefore, the JUPITER and CANTOS studies have highlighted the direct correlation between the reduction in the inflammatory state, specifically through the inflammasome pathway of the family containing leucine and pyrin domain-3 (NLRP3) and IL-1β [50,51], and the reduction in CV events of an atherosclerotic basis in healthy patients or in secondary prevention [52]. There are several signaling pathways associated with the inflammatory response; they have been implicated within atherosclerosis such as the NLRP3 inflammasome, Toll-like receptors, proprotein convertase subtilisin/kexin type 9, and the Notch and Wnt signaling pathways: all of these are important for atherosclerosis development and progression. Targeting inflammatory pathways such as the NLRP3 inflammasome pathway, and its regulated inflammatory cytokine interleukin-1β, represents a new method for the treatment of atherosclerotic diseases [53].

Other molecules that contribute to the inflammatory response are intercellular adhesion molecule (ICAM)-1, vascular cell adhesion molecule (VCAM)-1 and P-selectin. The L-, P- and E-selectin families of adhesion molecules contain an N-terminal lectin-like domain, followed by an epidermal growth factor (EGF)-like domain, and a variable number of repeating units with homology to the EGF complement/protein regulator. ICAM are expressed on endothelial, epithelial, fibroblast and leukocyte cells, and many tumor cells, and VCAMs are also widely distributed on endothelial, epithelial, macrophage and dendritic cells. ICAM-1s and VCAM-1s on endothelial cells appear to be particularly important for leukocyte attachment and transendothelial migration. The cell-surface expression of adhesion molecules such as ICAM-1s and VCAM-1s is upregulated in immune responses and inflammation, mediating cell activation and migration [54].

All of these considerations led us to explore further factors which, in parallel or even independently of an excess of blood lipids, can determine the onset of or aggravate this low-grade chronic inflammation which, over time, also affects the arterial vessels. We then specifically refer to the dysbiosis of the oral and the intestinal microbiota [55,56,57], the latter of which has already been linked to dyslipidemia, inflammation and ASCVD [58].

## 3. The Contribution of Oral Microbiota Dysbiosis to the Pathophysiology of Atherosclerotic Cardiovascular Diseases: Clinical Evidence and Molecular Mechanisms

In recent years, research has once again turned its interest towards the alterations of the microbial species living in our body—dysbiosis—in particular of the gastro-intestinal and oral microbiota. This pathogenic hypothesis has regained interest through numerous studies that have investigated the possible role of dysbiosis in the genesis or progression of the conditions that determine ASCVD.

The term “microbiome” was coined by researchers Whipps, Lewis and Cooke in 1988 to indicate a microbial community residing in a habitat that has well-defined chemical-physical properties [59]. Therefore, the “microbiota” fits into the biological context of this environment as the set of different living microorganisms that make up the microbiome [60]. The microbiota includes bacteria, archaea, fungi, protists and algae; phages, viruses, plasmids and free DNA, not being considered as living microorganisms, are rather contemplated in the microbiome [61]. The latter is finally completed by the so-called “theater of activity” of microorganisms, a term with which Whipple and collaborators defined the set of molecules and metabolites produced by the microbiota, structural components of the microbiota, molecules produced by the host organism itself and the aforementioned phages, viruses, mobile genetic elements and extracellular free DNA [59].

The study of the microbiome culminated in the “Human Microbiome Project” (HMP—Human Genome Project), promoted by the various National Institutes of Health of the United States of America (NIHs), which analyzed the microbiological niches of various body districts, in healthy subjects, by sequencing the 16S subunit of ribosomal RNA and high-throughput next-generation sequencing (nucleic acid sequencing) [62]. This project has highlighted how bacteria, among the viscera, cavities and integuments of our organism, are mainly distributed at the gastro-intestinal and oral level.

An aspect of great physiopathological relevance of the various microbiological niches consists of dysbiosis, that is, the modification of their ecological composition compared to an otherwise healthy subject [63]. There are several conditions that can alter the normal microbiota: the genetics of the host organism, diet, infections that have occurred, and medical intervention such as the use of antibiotics; these phenomena alter the normal balance between the species constituting the community, allowing some to grow at the expense of others and thus become virulent [63]. It follows that dysbiosis can not only be the consequence of a trigger that precedes them, but also the de novo cause of pathology or aggravation of a pre-existing status: in fact, in recent decades, numerous studies have documented significant changes in microbial communities of patients and animal models of inflammatory bowel disease [64], DM [65], asthma [66], allergies and even autism [67].

A recent important area of research is dysbiosis in the oral cavity. Indeed, low-grade bacteremia has been observed after tooth extraction, subgingival cleaning, endodontic treatment, third molar surgery or post-tonsillectomy [68,69]. With these premises, it is hypothesized that the same infection of the periodontal tissue can cause transient bacteremia, with direct bacterial invasion of the endothelial cells and CVD, or gastro-intestinal diseases, colorectal cancer, insulin resistance and diabetes mellitus, or even Alzheimer’s disease [70,71].

In fact, limited to the cardiovascular field, Mattila and collaborators in 1989 highlighted a large burden of most infectious dental pathological conditions, such as caries, periodontitis, periapical lesions or pericoronitis, in Scandinavian patients with a history of recent myocardial infarction rather than in healthy people [72]. The oral structures to be considered within this pathological context are the dental arches with their constituent elements: the tooth, the periodontium and the alveolar mucosa. The tooth is an organ housed within the alveolar processes of the mandible and maxillary bone and is made up of two superficial tissues, the enamel and the cementum, and two deep tissues, the dentin and the pulp [73]. Enamel and cement, respectively, cover the crown and the root (the latter is not visible). The pulp is the only soft tissue of the tooth and is housed inside the pulp cavity, in which the dental vessels and nerves flow: the pulp cavity extends from the crown to the root, which is the deepest portion of the tooth. At the root apex, the pulp communicates with the periodontal environment through an apical hole [73]. Around the tooth are its support structures, which surround it and stabilize it in the alveolar arch. They constitute the periodontium, characterized by hard tissues such as the cementum and the alveolar bone, and by soft tissues such as the periodontal ligament and gingiva, vascularized and innervated. Finally, the alveolar mucosa is the dentofugal anatomical continuation of the gingiva in its function of covering the alveolar bone [73]. The tooth is covered by a transparent film—biofilm—structurally and functionally organized, to which we refer with the term “dental plaque”. It consists of mostly acidogenic bacterial species immersed in a polymeric, glucidic, protein and aqueous matrix of bacterial and human derivation, and in healthy subjects it maintains its ecological characteristics in a rather stable manner. The plaque is able to favor the oral homeostasis of the host and, at the same time, defends the microbial community from harmful agents: the biological diversity inherent in it ensures the maintenance of these balances, preventing potentially pathogenic species from becoming virulent [74].

The study by Keijser and collaborators, by means of PCR analysis on salivary and dental plaque samples, has highlighted the prevalence of the following phyla: in the plaque there are mostly Actinobacteria, Fusobacteria and Spirochaetes, while in saliva Bacteroides, Firmicutes and Proteobacteria are prevalent [75]. In total there are about 800 microbial species that can form dental plaque and, of these, on average 150 are commonly found in each individual, including Gram-negative anaerobes [76].

Over the years, among the affections of the oral cavity, the importance of periodontitis (the inflammatory-infectious disease of the periodontium with polymicrobial etiology) has been particularly underlined. Periodontitis begins with gingivitis, or from a gingival inflammation due to the virulentation of a bacterial plaque that is not adequately managed by the individual through oral hygiene. Oral hygiene is the set of procedures designed to avoid the excessive growth of plaque, which can rather undergo mineralization and tenaciously adhere to the tooth itself. The growth of plaque occurs to the detriment of the normal groove between the tooth and the gum, generating the gingival pocket. As the pathological phenomenon becomes more severe, the periodontal structures are damaged and the systemic spread of bacterial-borne molecules can occur. The extent of the harmful process can even result in tooth loss. There are numerous risk factors for periodontitis: cigarette smoking, diabetes mellitus, immunosuppression, excessive accumulation of bacterial plaque, poor oral hygiene, genetic predisposition, pre-existing inflammation, and socio-economic factors. They contribute to the perturbation of the microbial biofilm, which causes dysbiosis and damage to the tooth structures; rather, the protective factors are the innate and adaptive immune response, host genetics, and integrity of the dental anatomy [76].

As already mentioned, a very important aspect of periodontal disease is the association between periodontitis and atherosclerotic disease. This relationship has been corroborated over the years by an ever-increasing amount of evidence. In addition to the studies mentioned above [72], the detection of bacterial material inside atherosclerotic plaques in vivo is of considerable interest: for example, Koren and collaborators have detected the presence of oral and intestinal pathogens such as Proteobacteria, Bacteroidetes, Firmicutes and Actinobacteria by PCR on samples obtained by post-endarterectomy atheromatous plaques from patients [29].

A 2013 review by Reyes and collaborators [77] aimed to evaluate the direct pro-atherogenic role of periodontal bacteria through seven evidence tests, each of which was commented on the basis of the pre-existing epidemiological, clinical and experimental literature. According to this evidence, oral bacteria (Table 3) are able to disseminate from the oral cavity into systemic circulation and reach the systemic vascular tissue. Numerous research groups have indeed demonstrated systemic bacterial dissemination, which could occur through multiple mechanisms. The most frequent condition results from manipulations in the oral cavity, both during daily life activities as well as in dental procedures [68]. Another proven mechanism is represented by transcellular-mediated, invading oral cells entering gingival microcapillaries in correspondence with the gingival pocket [78]. There is also an unproven theory, the “Trojan horse theory”, that proposes that pathogens can spread via phagocytosis mediated by circulating immune cells [79] and also escape from microbial killing [80]; these can be found in the affected vascular tissue. Samples of DNA, RNA or antigens from *Porphyromonas gingivalis* (*Pg*), *Actinobacillus actinomycetemcomitans* (*Aa*) [81] or *Veilonella* sp. [29] have been detected in atheromatous samples, concluding that multiple pathogenic species can reach the affected site. However, the results are conflicting and lacking in consistency due to the differences in methodology or according to the etiology of the atheroma [82], which reside, alive, within the affected tissue. For many years, research groups attempted to culture periodontal pathogenic microorganisms from atheromatous plaques without success, until 2005, when Kozarov and collaborators found the presence of vital *Pg* and *Aa* inside atheromatous tissue cultured with primary human coronary endothelial cells [83]; in vitro they invade affected tissue cells. The in vitro ability of periodontopathogens to invade cardiovascular cells is now established, especially for *Pg* [84]; this causes the promotion of atherosclerosis in animal models. Different animal models were used to support the hypothesis of a pathogenic role of *Pg* in atherosclerosis. In murine models, *Pg* has been reported as an important factor in accelerating the development of atherosclerosis [85]. In normocholesterolemic pigs, it induced aortic and coronary lesions, while in hypercholesterolemic pigs *Pg* bacteremia enhanced atherosclerosis [86]. Rabbits with periodontal disease developed fatty streaks in the aorta faster than controls [86]; non-invasive mutants of periodontal bacteria cause, in vitro and in vivo, a decidedly lower disease burden. *Pg* mutants with a reduced invasive ability have been demonstrated, in ApoE-gene-knockout mice (essential for HDL function), not to cause the acceleration of atherosclerosis, as opposed to the normo-invasive wild-type bacterial strain [87]; material isolated from human atheromas is capable of causing the disease in animal models of infection. This last aspect, which refers to Koch’s postulates, however, still needs to be proved. It requires the isolation and characterization of periodontopathogens from human atheromas and the demonstration that the isolates cause atherosclerotic pathology.

In this review, the advances in understanding the relationship between oral bacteria and atherosclerosis were recalled. Endothelial dysfunction can also be induced by infection [88]; chronic periodontal infection induces endothelial activation or dysfunction by a systemic inflammatory state evidenced by elevated plasma levels of acute phase protein, interleukin (IL)-6, and fibrinogen [89]. Systemic release of bacterial products may occur, such as membrane vesicles or Gingipains secreted by *Pg* [90] or soluble components released by *Aa* [91]. Proatherogenic dysfunction can be induced by direct bacterial invasion of the endothelium [77].

This review [92] analyzed the connection between lipid metabolism and the oral microbiome. *Pg* is able to promote pathological changes in lipid metabolism. In general, periodontal pathogens seem to have the ability to oxidize lipoproteins [93]. Kim et al. found that *Pg* induces the oxidation of high-density lipoproteins (HDL), impairing the atheroprotective function of these lipoproteins [94]. Furthermore, as indicated by Joo et al., *Pg* is characterized by a higher ability to oxidize low-density lipoprotein (LDL) than *C. pneumoniae* and *M. tuberculosis* [95]. *Pg* produces two different protease variants called Gingipain, capable of producing reactive oxygen species (ROS) and consuming antioxidants. Furthermore, the Gingipain Rgp and Kgp are able to induce lipid peroxidation [96]. Ljunggren et al. demonstrated that patients with PD have an altered plasma lipoprotein profile, also defined by altered post-translational protein levels and other structural modifications towards an atherogenic form [97].

In a study conducted by Reyes et al., *Pg* was found to invade and damage endothelial cells via ICAM-1 [98]. Furthermore, the invasion of gingival epithelium and endothelial cells by *Pg* may be facilitated by *F. nucleatum* and *T. forsythia* [99,100]. The infection causes direct damage to these cells, with mitochondrial fragmentation, an increase in ROS and a decrease in the concentration of adenosine triphosphate. Mitochondrial dysfunction is thought to be the mechanism by which *Pg* accelerates the progression of atherosclerosis [101]. It is additionally believed that *Pg* also affects the permeability of the vascular endothelium; Gingipain products, and especially Gingipain-K, are capable of inducing proteolytic cleavage of endothelial cell adhesion molecules-1 and VE-cadherin [102].

Finally, the clinical association between chronic periodontal infection and atherosclerosis was studied by Desvarieux and collaborators, through the INVEST Study, which evaluated groups of individuals over time starting from an initial cohort of 1056 subjects with no history of stroke or myocardial infarction, residing in Manhattan, New York, NY, USA. Objective dental evaluations were carried out with sampling of gingival plaque and ultrasound measurement of carotid-artery-wall thickness (intima-media thickness—IMT) as a morphological marker of progression of subclinical atherosclerosis.

A first finding from INVEST in 2003 evaluated the relationship between objective findings of periodontal disease with tooth loss and the presence of carotid atheromas, highlighting a greater prevalence of atherosclerotic plaque in patients with worse periodontal status (especially in the over 65 s [103]). In 2005, Desvarieux and collaborators, in the context of the INVEST study cohort, highlighted a positive relationship between IMT and increasing concentrations of odontopathogens at baseline, in particular *Pg* [104].

Subsequently, in 2013, the association between a clinical and microbiological improvement in the periodontium and less progression of IMT at 3 years of follow-up was highlighted [105].

Thus, periodontitis was found to be a significant risk factor for peripheral and carotid ASCVD in meta-analyses including large populations [55,56,57]. These observations suggest that pathological changes in oral microbiota composition and related periodontitis may play a role in the pathophysiology of ASCVD by promoting chronic inflammation, dyslipidemia, including LDL-C oxidation and reduced antiatherogenic high-density lipoprotein cholesterol (HDL-C) [106], endothelial cell dysfunction [107], and possibly other as-yet unknown disease processes [108]. In particular, the overall periodontal status of patients with periodontitis is mirrored by the relative abundance of total subgingival plaque-specific bacteria in the salivary microbiota [109], suggesting the reliability of sampling the latter biological matrix in this context.

## 4. Evaluation of Oral Health and Low-Grade Inflammation in Future CV Risk Detection Strategies

**Figure 2:**
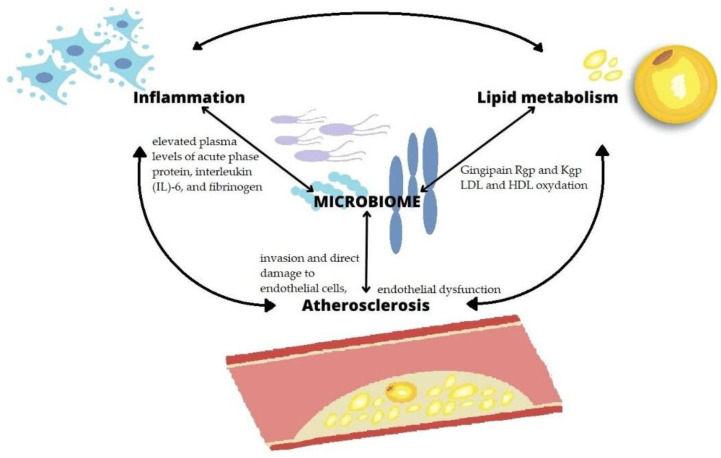
Interconnection between microbiome, inflammation, lipid metabolism and atherosclerosis.

This review has highlighted the interconnection existing between inflammatory status, lipid metabolism, oral microbiome and atherosclerosis, as shown in Figure 2.

The current evidence of a strong relationship between oral dysbiosis and related inflammatory conditions, and the promotion of ASCVD, as discussed in the present paper, has several important clinical implications. A general clinical consideration is that cross-communication between dentists and cardio-metabolic clinicians should be reinforced, leading the former to invite their patients to undergo a cardiovascular assessment, and, vice versa, the latter to take care of their oral health in the case of parodontitis and related conditions. A next step may be the concomitant assessment of oral health and low-grade chronic inflammation in the context of future CVR detection and reduction strategies.

The possibility of integrating oral microbiota sampling into clinical practice, in light of the growing support by the literature, could facilitate the evaluation of CVR and even modify the prognosis. To our knowledge, nowadays there are no reports on the specific abundance of the mentioned bacterial strains in the oral microbiota of patients at high risk of CVD in secondary CV prevention, with or without HeFH. In our study [110], we studied oral dysbiosis, i.e., the altered abundance of *Pg* and *Fn*, and its relationship to previous ASCVD in patients at high/very high risk for CVD, with or without HeFH, compared to matched healthy controls, finding that greater oral *Pg* abundance is present in very high-risk patients with previously diagnosed ASCVD and suggesting a potential relationship with CV events. On the other hand, even if at the moment the results are conflicting [45,46,47], insights on inflammatory markers more specific than hsCRP, such as, for example, the soluble urokinase plasminogen activator receptor (suPAR) [111], and new monitoring techniques will probably be the next innovation in CVR assessment.

## Figures and Tables

**Table 1:** Cardiovascular risk factors [4].

Cardiovascular Risk Factors	
Non modifiable risk factors	Modifiable risk factors
Age	Hypertension
Gender	Dyslipidemia
Genetic predisposition	Diabetes
Ethnicity	Obesity
	Smoking
	Alcohol abuse
	Unhealthy diet
	Physical inactivity
	Psychosocial factors

**Table 2:** The European Society of Cardiology (ESC) guidelines [13].

ASCVD Is Defined Based on the Presence, Clinically or Radiologically Documented
-a previous coronary event
-stable angina
-coronary revascularization (e.g., percutaneous coronary intervention—PCI)
-stroke or transient ischemic attack (TIA)
-peripheral artery disease
-significant coronary atherosclerotic plaque with a stenosis greater than 50% of the vessel diameter

**Table 3:** Link between periodontal disease and atherosclerosis.

The Link between Periodontal Disease and Atherosclerosis: Evidence and Mechanisms			Reference
1. Systemic dissemination of oral bacteria	Interventions in the oral cavity and bacterial dissemination	They can cause bacteremia leading to systemic inflammation and contributing to atherosclerosis development.	[68] Li X et al., Systemic diseases caused by oral infection.
	Immune cell-mediated bacterial dissemination	This mechanism can occur even in the absence of periodontal disease, as oral bacteria are constantly present in the oral cavity and can be taken up by immune cells.	[79] Carrion J. et al., Microbial carriage state of peripheral blood dendritic cells (DCs) in chronic periodontitis influences DC differentiation, atherogenic potential. [80] Zeituni A. E. et al., Targeting of DC-SIGN on human dendritic cells by minor fimbriated *Porphyromonas gingivalis* strains elicits a distinct effector T cell response.
	Gingival microcapillaries and bacterial dissemination	The gingival pocket provides a direct pathway for bacteria to enter the bloodstream, leading to systemic inflammation and contributing to the development of atherosclerosis.	[78] Takeuchi H. et al., Exit of intracellular *Porphyromonas gingivalis* from gingival epithelial cells is mediated by endocytic recycling pathway.
2. Presence of oral bacteria in atheromatous tissue	Association between periodontal disease and atherosclerosis	This association has been supported by numerous studies and suggests that periodontal disease may contribute to the development of atherosclerosis.	[82] Figuero E. et al., Detection of periodontal bacteria in atheromatous plaque by nested polymerase chain reaction.
	DNA, RNA, and antigen detection in atheromatous samples	Samples of atheromatous tissue have been found to contain DNA, RNA, and antigens from *Porphyromonas gingivalis*, *Actinobacillus actinomycetemcomitans*, and *Veilonella* sp.	[29] Omry Koren et al., Human oral, gut, and plaque microbiota in patients with atherosclerosis. [81] Haraszthy V. I. et al., Evidence for the role of highly leukotoxic *Actinobacillus actinomycetemcomitans* in the pathogenesis of localized juvenile and other forms of early onset periodontitis.
3. Survival of oral bacteria within atheromatous tissue	Mechanisms of survival of oral bacteria within atheromatous tissue	It has been suggested that oral bacteria may be able to evade the immune system and persist within the tissue by forming biofilms or by interacting with host cells.	[83] Kozarov E. et al., Human atherosclerotic plaque contains viable invasive *Porphyromonas gingivalis* and *Actinobacillus actinomycetemcomitans*.
	Presence of vital *Porphyromonas gingivalis* and *Actinobacillus actinomycetemcomitans* within atheromatous tissue	The presence of vital *Porphyromonas gingivalis* and *Actinobacillus actinomycetemcomitans* within atheromatous tissue provides evidence that these bacteria can survive within the affected tissue.	
	Culture of periodontal pathogenic microorganisms from atheromatous plaques	Vital *Porphyromonas gingivalis* and *Actinobacillus actinomycetemcomitans* were found inside atheromatous tissue cultured with primary human coronary endothelial cells.	
4. Invasion of oral bacteria into cells of affected tissue	Mechanisms of invasion of oral bacteria into cells of affected tissue	Oral bacteria may be able to use adhesins or other virulence factors to attach to host cells and then use secretion systems to inject effector proteins into the host cell, leading to invasion.	[84] Deshpande R. G. et al., Invasion of aortic and heart endothelial cells by *Porphyromonas gingivalis*.
	In vitro studies of *Porphyromonas gingivalis* invasion	Oral bacteria may be able to invade the cells of the affected tissue and contribute to the development of atherosclerosis.	
5. Induction of atherosclerosis by periodontal bacteria in animal models	Acceleration of atherosclerotic process in mouse models by *Porphyromonas gingivalis*	*Porphyromonas gingivalis* has been shown to accelerate the atherosclerotic process in mouse models.	[85] Lalla E. et al., Oral infection with a periodontal pathogen accelerates early atherosclerosis in apolipoprotein E-null mice.
	Induction of aortic and coronary lesions in normocholesterolemic pigs by *Porphyromonas gingivalis*	*Porphyromonas gingivalis* has been shown to induce aortic and coronary lesions in normocholesterolemic pigs.	[86] Jain A. et al., Role for periodontitis in the progression of lipid deposition in an animal model.
	Induction of atherosclerosis in hypercholesterolemic pigs following recurrent bacteremia by *Porphyromonas gingivalis*	*Porphyromonas gingivalis* has been shown to induce atherosclerosis in hypercholesterolemic pigs following recurrent bacteremia.	
6. Reduced disease burden with non-invasive mutants of periodontal bacteria	Reduced disease burden with non-invasive mutants of *Porphyromonas gingivalis*	*Porphyromonas gingivalis* mutants with reduced invasion ability do not cause acceleration of atherosclerosis in knockout mice for the Apo-E gene, which is essential for HDL function.	[87] Gibson F. C. 3rd et al., Innate immune recognition of invasive bacteria accelerates atherosclerosis in apolipoprotein E-deficient mice.
	Knockout mice for the Apo-E gene and acceleration of atherosclerosis	*Porphyromonas gingivalis* mutants with reduced invasion ability do not cause acceleration of atherosclerosis in these mice, suggesting that the invasion capacity of oral bacteria may be an important factor in their ability to contribute to the development of atherosclerosis.	
	Mechanisms of reduced disease burden with non-invasive mutants of periodontal bacteria	It has been suggested that reduced invasion ability may lead to reduced inflammation and immune activation, which may in turn lead to reduced atherosclerosis.	
7. Koch’s postulates and the causative role of oral bacteria in atherosclerosis	Koch’s postulates and the causative role of oral bacteria in atherosclerosis	The causative role of oral bacteria in atherosclerosis has not yet been established using Koch’s postulates.	-
	Material isolated from human atheromas and animal models of infection	Material isolated from human atheromas has been shown to be capable of causing the disease in animal models of infection.	
	Challenges in proving the causative role of oral bacteria in atherosclerosis	Proving the causative role of oral bacteria in atherosclerosis is challenging due to the complex nature of the disease and the multiple risk factors involved.	
